# Multi‐Omics Integration for Advancing Glioma Precision Medicine

**DOI:** 10.1002/acn3.70249

**Published:** 2025-11-17

**Authors:** Maria Guarnaccia, Valentina La Cognata, Giulia Gentile, Giovanna Morello, Sebastiano Cavallaro

**Affiliations:** ^1^ Institute for Biomedical Research and Innovation (IRIB) National Research Council (CNR) Catania Italy

**Keywords:** artificial intelligence, gliomas, multi‐omics strategies, personalized medicine, therapeutic interventions

## Abstract

Gliomas are among the most malignant and aggressive tumors of the central nervous system, characterized by the absence of early diagnostic markers, poor prognosis, and a lack of effective treatments. Advances in high‐throughput technologies have facilitated a refined molecular classification of gliomas, incorporating genetic features. However, diagnosis and clinical management based on isolated genetic data often fail to capture the full histological and molecular complexity of these tumors, posing significant challenges. In the era of computational methodologies and artificial intelligence, the integration of multiple omics layers—genomics, transcriptomics (including sex‐dependent differential expression patterns), epigenomics, proteomics, metabolomics, radiomics, single‐cell analysis, and spatial omics—into a comprehensive framework holds the potential to deepen our understanding of glioma biology and enhance diagnostic precision, prognostic accuracy, and treatment efficacy. Herein, we provide a comprehensive overview of multi‐omics strategies used to decipher the adult‐type diffuse glioma molecular taxonomy and describe how the integration of multilayer data combined with machine‐learning‐based algorithms is paving the way for advancements in patient prognosis and the development of personalized, targeted therapeutic interventions.

**Abbreviations**
CL: classical
CNS: central nervous system
CNVs: copy number variants
CT: computed tomography
DME: DNA methylation‐expressed
EGFR: epidermal growth factor receptor
GBM: glioblastoma
LGG: low‐grade gliomas
MES: mesenchymal
MRI: magnetic resonance imaging
OS: overall survival
OXPHOS: oxidative phosphorylation
pd‐GBSC: patient‐derived GBM stem cell
PET: positron emission tomography
PN: proneural
scRNA‐seq: single‐cell transcriptomics
SNVs: single nucleotide variants
SPHINKS: Substrate PHosphosite‐based Inference for Network of KinaseS
TCGA: The Cancer Genome Atlas
VEGF: vascular endothelial growth factor
WHO: World Health Organization

## 1 Introduction

Gliomas are the most common primary brain tumors arising from glial cells of the central nervous system (CNS), with a global incidence of approximately 3.8 to 4.4 cases per 100,000 people annually for all gliomas (24,820 new cases in the US), and an incidence of about 3.3 to 3.9 per 100,000 for glioblastoma (12,000 new cases in the US), the most common and deadly glioma subtype [1]. Known by their infiltrative nature and heterogeneity, these types of malignancies span a broad spectrum of conditions classified into four grades of malignancy according to the CNS World Health Organization (WHO) system, each presenting distinct diagnostic and therapeutic challenges [2].

Clinically, gliomas manifest through a wide range of symptoms, often dictated by their location in the brain, including headaches, seizures, focal neurological deficits, and cognitive impairments [3, 4]. Diagnosis relies on a combination of neuroimaging techniques—magnetic resonance imaging (MRI), computed tomography (CT) and positron emission tomography (PET)—complemented by histopathological analysis obtained through surgical biopsy or tumor resection [5, 6]. Treatment strategies embrace a multidisciplinary approach, incorporating surgical resection to reduce tumor burden and relieve mass effect, along with adjuvant therapies such as radiotherapy and chemotherapy to target residual tumor cells and mitigate disease recurrence [7]. The advent of molecularly targeted agents, such as the inhibitors of the epidermal growth factor receptor (EGFR) and vascular endothelial growth factor (VEGF), is providing some innovative avenues for tailored treatment selection and personalized therapeutic interventions [8, 9], offering both challenges and opportunities in accurately diagnosing, classifying, and managing gliomas.

The emergence of Omics Sciences and the development of The Cancer Genome Atlas (TCGA) have significantly improved the ability to distinguish different layers of glioma heterogeneity, leading to a revised WHO classification system that integrates multiple molecular profiles, including DNA methylation patterns, gene expression signatures, and mutational landscapes. According to the latest classification, the prognosis and treatment of gliomas vary significantly depending on the glioma type—such as astrocytoma, IDH‐mutant (grades 2, 3, or 4), oligodendroglioma, IDH‐mutant with 1p/19q codeletion (grades 2 or 3), and glioblastoma, IDH‐wildtype (grade 4)—as well as on molecular markers including IDH1/2, H3‐3A, ATRX, CDKN2A/B, 1p/19q codeletion, TERT promoter mutations, MGMT promoter methylation, EGFR amplifications, PTEN deletions, and BRAF alterations [10, 11].

**FIGURE 1:**
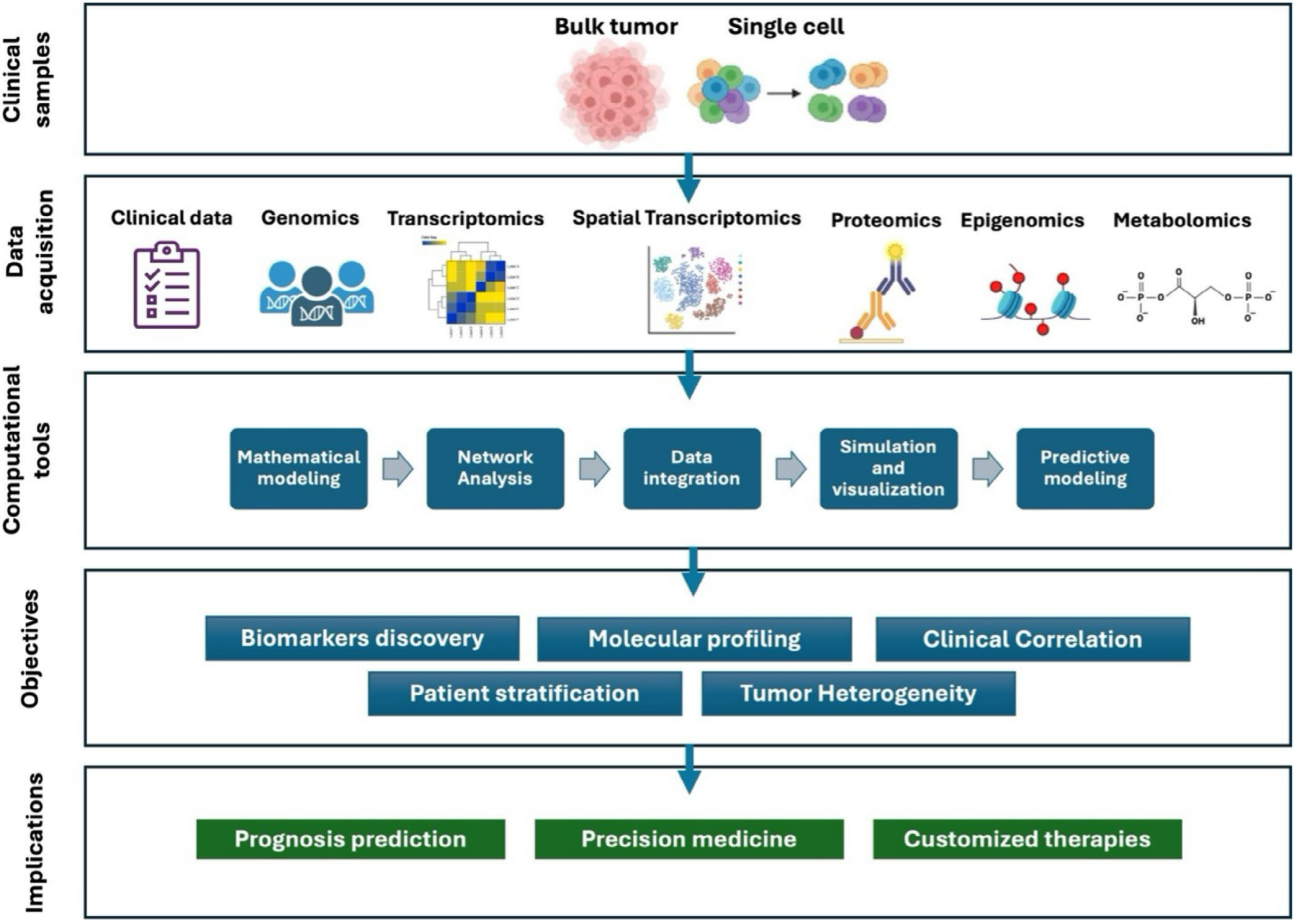
Integrative multi‐omics and network systems modeling in gliomas. Schematic representation of how multi‐omics datasets can be integrated by computational tools to achieve a comprehensive understanding of glioma biology. This approach enables the identification of key molecular drivers, subtype‐specific vulnerabilities, prognostic biomarkers, and potential therapeutic targets.

While single‐omics approaches offer a limited view that fails to fully capture the tumor's diverse histological, cellular, and molecular characteristics, modern computational methods and artificial intelligence‐driven technologies are facilitating the integration of multiple omics layers—genomics, transcriptomics, epigenomics, proteomics, metabolomics, radiomics, and single‐cell analysis—providing a holistic perspective on glioma biology. This multidimensional approach enhances diagnostic precision, refines prognostic predictions, and improves therapeutic outcomes (Figure 1) [12, 13, 14]. The growing adoption of integrative omics strategies is uncovering previously unknown molecular relationships, pathways, and biological processes. The linkage of these findings with patient‐specific clinical data is emerging as a critical step toward identifying molecular signatures that hold significant potential for personalized medicine and individualized treatment approaches (Figure 1).

In this work, we provide a comprehensive overview of the role of multi‐omics methodologies in glioma research and explore how the integration of multilayered data is advancing glioma taxonomy, prognosis and personalized therapeutic strategies.

## 2 Molecular Classification of Adult Type Diffuse Gliomas: State of Art

Accurately grading gliomas remains a significant challenge, as their pathogenesis results from the accumulation of malignant genetic alterations that define distinct molecular subtypes associated with specific clinical features [15, 16, 17]. Imaging plays a pivotal role in glioma diagnosis, with contrast‐enhanced CT and MRI serving as the primary modalities for initial assessment and staging [18]. CT is commonly utilized in emergency settings as the first‐line imaging tool to rapidly evaluate brain lesions, hemorrhages, calcifications, and acute complications [19], whereas MRI provides detailed visualization of tumor boundaries, edema, and interactions with surrounding brain tissue [20]. Advanced MRI techniques further enhance diagnostic precision by offering integrated anatomical, functional, and metabolic insights [21].

**FIGURE 2:**
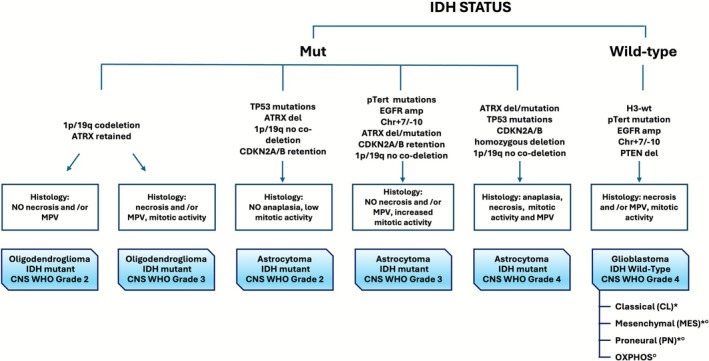
The 2021 WHO classification of gliomas, integrating histopathological features and molecular markers. MPV, microvascular proliferation; transcriptional subtypes (*); patient‐derived glioma cell lines subtypes (°).

The integration of traditional histopathological criteria with genetic and molecular biomarkers has recently reshaped glioma classification, advancing toward a personalized medicine approach. The revised WHO 2021 classification (Figure 2) had a profound impact on clinical management, categorizing primary gliomas into grades 1 to 4, corresponding to low‐grade gliomas (LGG) and high‐grade gliomas (HGG). Specifically, these include: Pilocytic astrocytoma (CNS WHO grade 1); Oligodendroglioma, *IDH*‐mutant and 1p/19q‐codeleted (CNS WHO grades 2 and 3); Astrocytoma, *IDH*‐mutant (CNS WHO grade 3); Astrocytoma, *IDH*‐mutant with *CDKN2A/B* homozygous deletion (CNS WHO grade 4); Glioblastoma, *IDH*‐wildtype (CNS WHO grade 4); and Glioblastoma, *IDH*‐wildtype with *TERT* promoter mutation, *EGFR* amplification, or chromosome 7 gain/chromosome 10 loss (CNS WHO grade 4) [22, 23].

Moreover, glioblastoma (GBM) is further classified into three transcriptional subtypes—proneural (PN), mesenchymal (MES) and classical (CL)—or, in patient‐derived glioma cell line models, into PN, MES and OXPHOS subtypes, each characterized by distinct molecular alterations, therapeutic sensitivities, and prognostic implications [24, 25]. Recent studies have also described hybrid and transitional glioma subtypes, exhibiting mixed transcriptional profiles and intermediate phenotypes [26], highlighting the dynamic nature of glioma biology and the existence of transitional states between molecular subgroups [27].

**FIGURE 3:**
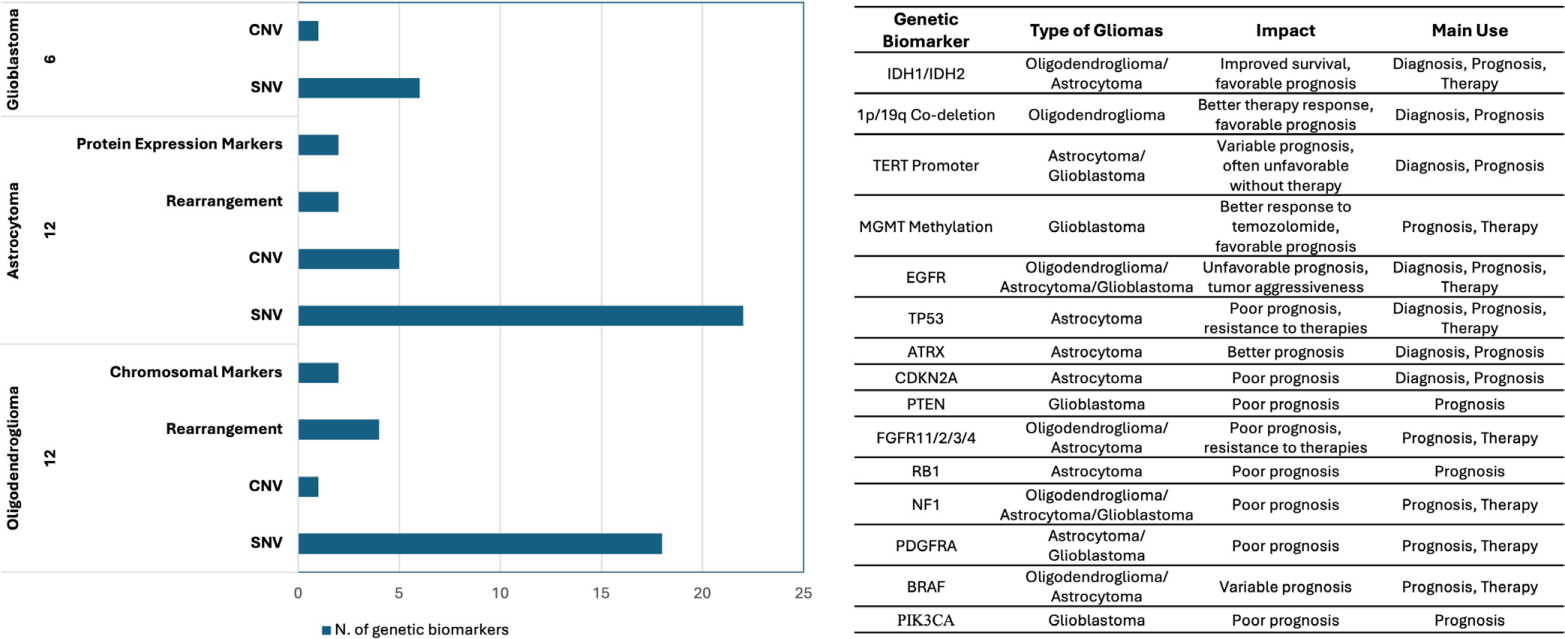
Glioma genetic biomarkers from “My Cancer Genome”. The histogram displays genetic biomarkers in different glioma types currently listed in “My Cancer Genome” (accessed October 2025). The accompanying table summarizes the key genetic biomarkers utilized for diagnosis, prognosis or treatment in specific glioma subtypes.

Numerous investigations have identified additional genetic alterations and biological markers that inform tumor classification [28, 29, 30, 31, 32], glioma differentiation, disease progress estimation [33], patient survival assessment and therapeutic response prediction [2, 34, 35]. Currently, the “My Cancer Genome” tool (https://www.mycancergenome.org) catalogs a broad spectrum of genetic biomarkers—including Single Nucleotide Variants (SNVs), Copy Number Variants (CNVs), structural rearrangements, protein expression markers, chromosomal aberrations and genomic instability indicators—associated with different glioma types. Many of these biomarkers are actively used for diagnosis, prognosis and treatment selection and are linked to ongoing clinical trials [36, 37] (Figure 3).

The molecular markers of each glioma subtype further influence prognosis and are critical for guiding treatment strategies. In LGG, key alterations include *IDH1/2*, *TP53* and *ATRX* mutations in Astrocytoma and 1p/19q co‐deletions in Oligodendroglioma. Generally, *IDH* mutation and retained *ATRX* expression correlate with better survival, whereas *TP53* mutations are often associated with poorer clinical outcomes, including therapy resistance and reduced survival. The presence of 1p/19q co‐deletion strongly predicts a favorable prognosis [38, 39].

In HGG, *IDH* mutations are also linked to improved prognosis and longer survival, whereas *CDKN2A* deletions, Histone H3 and *TP53* mutations are associated with poor outcomes [23]. *TERT* promoter (TERTp) alterations typically indicate a higher risk of recurrence and worse survival in both IDH‐wildtype and IDH‐mutant patients. However, when TERTp alterations co‐occur with *IDH* mutations, 1p/19q co‐deletion, and *MGMT* promoter methylation, they are associated with a favorable prognosis [40, 41]. *EGFR* alterations, particularly amplifications, generally predict worse survival in astrocytomas and glioblastomas, while *PTEN* alterations (mutations or deletions) are linked to poor prognosis specifically in GBM [42, 43].

## 3 Integrative Multi‐Omics Data for Glioma Molecular Taxonomy and Diagnosis

The integration of extensive biological data across multiple omics layers provides significant opportunities to refine our understanding of glioma pathogenesis and enhances diagnostic accuracy [44, 45, 46]. A range of computational tools and bioinformatics resources have been developed to support glioma diagnosis and research. For example, the *GlioMarker Database* offers clinicians and researchers access to glioma diagnostic biomarkers, clinical data, and genomic expression profiles [47]. The *IPD‐Brain Dataset* provides histopathological images with detailed clinical annotations and is frequently used to train deep‐learning models for glioma subtype classification and biomarker prediction [48]. *Glioma‐BioDP* consolidates gene, protein, histological, surgical status and survival data from low‐ and high‐grade glioma patient samples within the TCGA database [49]. Another valuable resource, the *Surveillance Epidemiology and End Results (SEER)* database provides population‐based cancer statistics for glioma risk prediction and treatment evaluation [50].

Several studies have demonstrated the utility of integrating genomic, epigenomic, and transcriptomic data with histopathological and clinical datasets to improve glioma prognosis predictions. For instance, Wu et al. developed a risk score model based on DNA methylation‐expressed (DME) gene profiles from 259 glioma samples (WHO grades I–III, TCGA database) and identified six DME genes (*EMP3, DDIT4L, MEOX2, OCIAD2, TGFB2, TNFRSF12A*) associated with survival outcomes [51]. Similarly, Munquad et al. applied deep‐learning approaches to transcriptomic and methylome data from TCGA glioma patients, achieving 98.03% accuracy in predicting LGG (astrocytoma, oligoastrocytoma, oligodendroglioma) and GBM (classical, mesenchymal, proneural) subtypes, with sensitivity above 92% [52, 53]. Binder et al. further employed Self‐Organizing Maps (SOM) to classify LGG samples into distinct clusters based on integrated omics layers, revealing unique biological characteristics [46].

More recently, Vieira et al. combined mRNA, DNA methylation, and miRNA data from TCGA, using the DIABLO method, a supervised sparse canonical correlation analysis approach, to identify highly correlated molecular features distinguishing GBM from LGGs. These analyses linked GBM to receptor tyrosine kinase signaling disruptions and extracellular matrix remodeling [54]. Other studies have leveraged omics integration to uncover biomarkers associated with inflammation, mitochondrial signaling, angiogenesis, and metabolism, as well as miRNA and metabolites implicated in GBM progression [55]. Additionally, the integration of transcriptomic, miRNA, and DNA methylation datasets has enabled LGG clustering according to distinct survival trajectories [56].

Further research has uncovered novel subgroups within oligodendroglial tumors, displaying varying levels of clinical and molecular aggressiveness [57]. Large‐scale multi‐omics integration studies by Jang et al. identified sex‐specific genetic determinants in GBM, uncovering differences in enriched signaling pathways, treatment responses, and overall survival (OS) [58, 59]. Finally, AI‐driven deep‐learning frameworks and tensor analysis techniques have been employed to extract key glioma biomarkers and stratify patients based on survival outcomes [60].

## 4 Integrative Multi‐Omics Approaches for Glioma Prognosis Prediction

The integration of omics data with artificial intelligence (AI) and machine learning (ML) algorithms is driving advances in predicting glioma prognosis. By employing a multi‐omics framework, researchers have developed models that incorporate genomic, transcriptomic, epigenomic, proteomic, and clinical data to enhance survival prediction accuracy.

For instance, Yang et al. constructed a multivariate Cox proportional hazards model for GBM and LGG patients, integrating five feature types: genetic, cytogenetic, transcriptomic, demographic, and pathologic variables. Their findings indicated that transcriptomic data served as the most informative molecular predictor of survival, while combining all variables achieved the highest accuracy [61]. Similarly, Wang et al. designed a recurrence‐related signature using multi‐omics data from primary and recurrent gliomas [62]. By applying LASSO regression to 108 prognostic genes, they identified an 18‐gene signature that stratified patients into high‐ and low‐risk groups, with the high‐risk group exhibiting shorter overall survival (OS) and earlier recurrence [62]. Other integrative models have incorporated pseudouridine levels [63], immune‐associated markers [64], and cell death profiles [65] for prognosis prediction.

Predicting survival in LGGs is particularly challenging due to their variable clinical trajectories, ranging from stable disease to rapid progression into GBM. To address this, Choi and Lee introduced Multi‐Prognosis Estimation Network (Multi‐PEN), a deep‐learning model integrating multi‐omics and multi‐modal data. This approach identified *MYBL1* and hsa‐mir‐421 as key prognostic markers, with high *MYBL1* expression and elevated miR‐421 correlating with favorable outcomes [66, 67, 68]. Consequently, *MYBL1* has been incorporated as a biomarker in the 2021 WHO classification. Additionally, Du et al. combined whole‐exome sequencing (WES), RNA sequencing, and DNA methylation analysis to develop a multi‐omics model distinguishing LGG patients with shorter versus longer survival in both training and validation cohorts [12]. Cao et al. introduced GInLncSig, a genomic stability‐based signature derived from somatic mutation and long noncoding RNA (lncRNA) data, identifying five lncRNAs whose lower risk scores predicted favorable prognosis in LGG patients [69, 70]. Similarly, Yang et al. integrated DNA methylation, copy number variation, and miRNA expression to classify LGGs into four molecularly distinct clusters (IC1–IC4), each exhibiting unique immune‐related signatures, prognostic trajectories, and clinical‐genetic correlations [71]. Despite histological similarity, these clusters differ in 1p/19q co‐deletion (IC1 and IC2), *MGMT* promoter methylation (IC2), *TERT* status, and mutational spectra of *IDH1*, *TP53*, and *EGFR*, translating into distinct survival outcomes [71].

Prognostic modeling of GBM remains particularly complex due to its aggressive progression and poor survival. Nassani et al. integrated gene expression, proteomics, and clinical metrics to identify molecular features associated with extended survival (6 months vs. 2 years) and improved Karnofsky performance scores (KPS). Using an iterative random forest (iRF) algorithm, they identified 35 molecular features (19 genes and 16 proteins) linked to GBM prognosis [72]. More recently, multi‐omics models combining histopathological image features with genomics, transcriptomics, and proteomics achieved high predictive performance for OS, with AUCs up to 0.926 for 3‐year survival prediction, demonstrating robust risk stratification capabilities [73].

## 5 Integrative Multi‐Omics Approaches for Personalized Targeted Treatment of Gliomas

Despite the extensive molecular characterizations of gliomas, substantial therapeutic improvements for patient outcomes remain elusive. However, integrating multi‐omics data is unlocking new opportunities for personalized medicine, allowing the identification of specific molecular alterations that can guide disease management.

Migliozzi et al. developed Substrate PHosphosite‐based Inference for Network of KinaseS (SPHINKS), a machine‐learning algorithm integrating proteomics and phospho‐proteomics data into a single network. SPHINKS enables unbiased identification of subtype‐specific master kinases driving glioblastoma phenotypes. In subtype‐matched GBM organoids, PKCδ and DNA‐PKcs were validated as master kinases for the glycolytic/plurimetabolic (GPM) and proliferative/progenitor (PPR) subtypes, highlighting them as promising therapeutic targets [74].

Another innovative approach, i‐Modern, is an integrated multi‐omics deep learning network capable of accurately stratifying patients into high‐risk and low‐risk subgroups. Leveraging mRNA, miRNA, DNA methylation, somatic mutations, CNVs, and protein expression data, i‐Modern identifies survival‐associated molecular signatures while reducing high‐dimensional data to informative features [75]. Top‐ranked signatures included 10 gene expression, 29 CNV, 29 methylation, 9 protein, and 3 miRNA signatures, highlighting therapeutic targets such as CD276 and TGFB1 [75].

Santamarina‐Ojeda et al. performed genome‐wide transcriptomic and DNA methylation analyses on bulk GBM tumors and patient‐derived GBM stem cells (pd‐GBSCs) [76]. Integrated analyses revealed distinct pathways regulated by *AP‐1*, *SMAD3*, and *RUNX1/2*, and inhibiting these pathways—alone or combined with temozolomide—significantly impaired tumor growth, particularly in aggressive mesenchymal‐like GBM [76].

Wu et al. integrated genomic, transcriptomic, and drug response data to classify patient‐derived glioma cell lines (PDGCs) into MES, PN, and OXPHOS subtypes, preserving key GBM driver alterations [77]. Drug screening for 214 FDA‐approved compounds revealed subtype‐specific vulnerabilities: PN was sensitive to tyrosine kinase inhibitors, OXPHOS to histone deacetylase inhibitors, oxidative phosphorylation inhibitors, and HMG‐CoA reductase inhibitors, while MES exhibited higher drug resistance [77].

Aberrant glycosylation contributes to tumor progression, metastasis, and therapy resistance. Strategies targeting glycosylation, using lectins, sialoglycans, glycosyltransferase inhibitors, and glycosidase inhibitors, are emerging to enhance immunotherapy efficacy [78, 79]. Forty‐four glycosylation regulators have been associated with glioma survival, and seven genes were linked to high‐risk patients. Glycosylation‐related targets are being investigated for diagnosis (PSCA, GnT‐III, sPTPRZ, Galectin‐3) and prognosis (TXNDC12, PLAUR, GALNT), while glycosylated proteins such as PTPRZ, dg‐Bcan‐targeting peptide (BTP), and nucleolin gp273 represent potential therapeutic targets. In LGG patients, aberrant glycosylation in eight genes demonstrated predictive value for prognosis and therapy response [80, 81].

Several clinical trials (SHIVA, MOSCATO 01, ProfiLER, I‐PREDICT, WINTHER) evaluated matched versus unmatched therapy based on next‐generation sequencing and comparative genomic hybridization. Some studies reported limited benefits due to unavailable drugs or insufficient modeling of tumor biology, while others demonstrated improved OS and progression‐free survival [82]. Yi Ding et al., developed a GloMICS prognostic model, analyzing ECM‐related genes across CGGA and TCGA‐GBM datasets to predict OS. This model accurately predicted survival at multiple time points and can guide personalized treatment decisions [83]. Yang et al. applied single‐cell RNA‐seq and snATAC‐seq to identify a mesenchymal GBM subtype with poor prognosis, predicting sensitivity to Trametinib and Dasatinib, validated in vitro [84]. A 2025 study identified AEBP1 and EFEMP2 as regulators of immune heterogeneity in GBM, informing immunotherapy strategies [85]. AI‐driven frameworks now integrate genomic, transcriptomic, epigenomic, and radiomic data for subtype classification and drug prioritization, exemplifying the transformative potential of multi‐omics in glioma precision medicine [86].

Collectively, these approaches demonstrate that multi‐omics integration can identify cancer cell vulnerabilities, stratify patients for targeted therapies, and advance the precision medicine paradigm in glioma treatment.

## 6 Spatial Omics as an Additional Informative Layer in Gliomas

Traditional bulk molecular techniques have been instrumental in advancing our understanding of glioma etiopathogenesis [87]. Each omics layer provides unique insights into cellular functions, helping to unravel disease mechanisms, identify driver mutations, and stratify patients based on molecular profiles. However, these approaches often fail to capture tumor heterogeneity, spatial organization, and cell–cell interactions, limiting the ability to assess dynamic processes within the tumor microenvironment [88].

In recent years, single‐cell transcriptomics (scRNA‐seq) has revolutionized cancer research, including glioma biology, by providing unprecedented resolution and depth [89]. This technology enabled the identification of multiple transcriptomic subtypes within individual tumors, detection of metastatic subpopulations, and discovery of predictive biomarkers, thereby facilitating drug response assessment and guiding treatment strategies [90]. Alongside scRNA‐seq, spatial transcriptomics has emerged as a powerful tool for mapping the distribution patterns of gene expression and cellular interactions within the tumor microenvironment [91, 92]. Together, these methods allow the identification of spatially distinct cellular subpopulations, niche‐specific gene expression signatures, and localized signaling pathways, offering a comprehensive view of tumor architecture and its interactions with neighboring cells and extracellular matrix [14, 93].

Although still in its early stages, the integration of single‐cell and spatial transcriptomics is gaining momentum [92]. A recent integrated analysis of 201,986 human glioma cells revealed extensive intra‐ and inter‐tumoral heterogeneity in immune cell composition across tumor regions and patients [88]. Both GBM and *IDH1*‐mutant gliomas exhibited pronounced variability, reflecting diverse immune landscapes within spatially distinct regions [94].

In GBM, Ravi et al. identified five spatially distinct transcriptional programs: radial‐glia, reactive‐immune, neural development, spatial oligodendrocyte precursor cell and reactive‐hypoxia, each associated with unique genomic alterations and shared transcriptomic signatures [95]. For example, the radial glia program is characterized by high expression of radial‐glia‐associated genes, such as *HOPX* and *PTPRZ1*, and astrocyte‐related genes (*GFAP, AQP4, VIM*, and *CD44*). The reactive‐immune program shows enrichment in inflammation‐associated genes (*HLA‐DRA, C3, CCL4*, and *CCL3*) and features interferon‐γ signaling. The neural development and oligodendrocyte precursor programs align with neuronal and oligodendrocyte lineages, respectively. The reactive‐hypoxia program involves hypoxia‐response genes (*VEGFR*, *HMOX1, GAPDH*) and glycolytic genes (*LDHA* and *PGK1*) [95].

Similarly, Ren et al. defined four gene expression patterns (invasive, hypoxic, vascular and tumor core), with corresponding niche‐specific markers [96].

Spatial transcriptomics has also facilitated the identification of therapeutic targets enriched in specific tumor regions, including *TP53, EGFR, FERMT1, CD44, S100A4*, and *SOX2*, particularly in blood vessel‐rich and tumor‐dense areas of high‐grade gliomas (*IDH* wild‐type and mutant) [97]. Yixin Fu et al. demonstrated distinct metabolic profiles across tumor regions: hypoxic cores rely on glycolysis and lactate metabolism (Warburg effect), whereas peripheral regions preferentially utilize glutamine metabolism and fatty acid oxidation [98].

These advancements position spatial‐omics as a crucial layer in integrative multi‐omics approaches, complementing molecular, genomic, and transcriptomic data. Moreover, spatially resolved molecular information aligns with the evolving paradigm of network‐preserving neurosurgery. By linking region‐specific tumor heterogeneity with functional brain circuits, spatial omics can inform surgical planning, guiding resection strategies to minimize disruption of critical networks while targeting the most aggressive tumor regions. As highlighted by Zhao J. et al. [99], multimodal intelligent neurological imaging combined with minimally invasive surgical techniques enables precise tumor targeting while protecting and remodeling functional brain networks [99, 100]. Integration of omics data, neurobiology, advanced imaging, surgical technology, and computational modeling represents a paradigm shift toward precision surgery, balancing tumor control with preservation of brain network integrity and optimizing patient prognosis.

## 7 Conclusions

Gliomas remain a formidable challenge in neuro‐oncology due to their infiltrative behavior, molecular heterogeneity, and therapeutic resistance [101]. Advances in molecular profiling have led to the identification of novel genetic markers and transcriptional signatures, refining glioma classification and enhancing clinical management [102].

The rapid evolution of multi‐omics data integration, combined with AI‐driven analytical methods, is increasingly contributing to accurate tumor classification, patient outcome prediction, and optimized treatment selection [103, 104, 105, 106]. AI‐enhanced algorithms now facilitate the integration of imaging modalities (X‐rays, MRIs, CT scans) and histopathological data with multi‐omics datasets, creating a more comprehensive diagnostic and prognostic framework.

Despite these advances, challenges remain. Technical variability, sample heterogeneity, and data interpretation complexities can affect the reliability and reproducibility of molecular findings [107]. These issues are particularly evident in gene expression studies, where differences in platforms, study design, sex‐dependent expression, and the dynamic nature of gene regulation necessitate longitudinal studies and functional validation to establish causal relationships between molecular alterations and glioma pathogenesis.

Nevertheless, ongoing research continues to yield promising results, supporting further investment in multidisciplinary omics approaches. As these strategies advance toward clinical translation, they hold the potential to enhance precision medicine and deliver meaningful therapeutic benefits to glioma patients.

## Author Contributions

Conceptualization: G.M., V.L.C., S.C.; Writing – original draft preparation: G.M., V.L.C.; Writing – review and editing: G.M., V.L.C., G.G., G.M.; Supervision: S.C.; Funding acquisition: S.C.

## Conflicts of Interest

The authors declare no conflicts of interest.

## Data Availability

Data sharing not applicable to this article as no datasets were generated or analyzed during the current study.

## References

[1] X. Guo , Y. Shi , D. Liu , et al., “Clinical Updates on Gliomas and Implications of the 5th Edition of the WHO Classification of Central Nervous System Tumors,” Frontiers in Oncology 13 (2023): 1131642. 36998447 10.3389/fonc.2023.1131642 PMC10043404

[2] F. Sahm , S. Brandner , L. Bertero , et al., “Molecular Diagnostic Tools for the World Health Organization (WHO) 2021 Classification of Gliomas, Glioneuronal and Neuronal Tumors; an EANO Guideline,” Neuro‐Oncology 25, no. 10 (2023): 1731–1749. 37279174 10.1093/neuonc/noad100 PMC10547522

[3] A. Sharma and J. J. Graber , “Overview of Prognostic Factors in Adult Gliomas,” Annals of Palliative Medicine 10, no. 1 (2021): 863–874. 32787379 10.21037/apm-20-640

[4] T. S. Armstrong , A. M. Bishof , P. D. Brown , M. Klein , M. J. Taphoorn , and C. Theodore‐Oklota , “Determining Priority Signs and Symptoms for Use as Clinical Outcomes Assessments in Trials Including Patients With Malignant Gliomas: Panel 1 Report,” Neuro‐Oncology 18, no. Suppl 2 (2016): ii1–ii12. 26989127 10.1093/neuonc/nov267 PMC4795996

[5] E. Ruiz‐Lopez , J. Calatayud‐Perez , I. Castells‐Yus , et al., “Diagnosis of Glioblastoma by Immuno‐Positron Emission Tomography,” Cancers (Basel) 14, no. 1 (2021): 74. 35008238 10.3390/cancers14010074 PMC8750680

[6] A. V. Bonm , R. Ritterbusch , P. Throckmorton , and J. J. Graber , “Clinical Imaging for Diagnostic Challenges in the Management of Gliomas: A Review,” Journal of Neuroimaging 30, no. 2 (2020): 139–145. 31925884 10.1111/jon.12687 PMC8300867

[7] G. Thenuwara , J. Curtin , and F. Tian , “Advances in Diagnostic Tools and Therapeutic Approaches for Gliomas: A Comprehensive Review,” Sensors (Basel) 23, no. 24 (2023): 9842. 38139688 10.3390/s23249842 PMC10747598

[8] S. Sathornsumetee , D. A. Reardon , A. Desjardins , J. A. Quinn , J. J. Vredenburgh , and J. N. Rich , “Molecularly Targeted Therapy for Malignant Glioma,” Cancer 110, no. 1 (2007): 13–24. 17520692 10.1002/cncr.22741

[9] L. Muzyka , N. K. Goff , N. Choudhary , and M. T. Koltz , “Systematic Review of Molecular Targeted Therapies for Adult‐Type Diffuse Glioma: An Analysis of Clinical and Laboratory Studies,” International Journal of Molecular Sciences 24, no. 13 (2023): 10456. 37445633 10.3390/ijms241310456 PMC10341773

[10] A. Penkova , O. Kuziakova , V. Gulaia , et al., “Comprehensive Clinical Assays for Molecular Diagnostics of Gliomas: The Current State and Future Prospects,” Frontiers in Molecular Biosciences 10 (2023): 1216102. 37908227 10.3389/fmolb.2023.1216102 PMC10613994

[11] H. S. Ghosh , R. V. Patel , E. Woodward , et al., “Contemporary Prognostic Signatures and Refined Risk Stratification of Gliomas: An Analysis of 4400 Tumors,” Neuro‐Oncology 27, no. 1 (2025): 195–208. 39164213 10.1093/neuonc/noae164 PMC11726335

[12] Z. Du , Y. Jiang , Y. Yang , et al., “A Multi‐Omics Analysis‐Based Model to Predict the Prognosis of Low‐Grade Gliomas,” Scientific Reports 14, no. 1 (2024): 9427. 38658591 10.1038/s41598-024-58434-8 PMC11043340

[13] V. Costa , C. Angelini , I. De Feis , and A. Ciccodicola , “Uncovering the Complexity of Transcriptomes With RNA‐Seq,” Journal of Biomedicine and Biotechnology 2010 (2010): 853916. 20625424 10.1155/2010/853916 PMC2896904

[14] R. Arora , C. Cao , M. Kumar , et al., “Spatial Transcriptomics Reveals Distinct and Conserved Tumor Core and Edge Architectures That Predict Survival and Targeted Therapy Response,” Nature Communications 14, no. 1 (2023): 5029. 10.1038/s41467-023-40271-4 PMC10439131 37596273

[15] S. Wang , F. Jin , W. Fan , et al., “Gene Expression Meta‐Analysis in Diffuse Low‐Grade Glioma and the Corresponding Histological Subtypes,” Scientific Reports 7, no. 1 (2017): 11741. 28924174 10.1038/s41598-017-12087-y PMC5603565

[16] F. Zeng , X. Liu , K. Wang , Z. Zhao , and G. Li , “Transcriptomic Profiling Identifies a DNA Repair‐Related Signature as a Novel Prognostic Marker in Lower Grade Gliomas,” Cancer Epidemiology, Biomarkers & Prevention 28, no. 12 (2019): 2079–2086. 10.1158/1055-9965.EPI-19-0740 31533943

[17] P. M. H. Tran , L. K. H. Tran , J. Nechtman , et al., “Comparative Analysis of Transcriptomic Profile, Histology, and IDH Mutation for Classification of Gliomas,” Scientific Reports 10, no. 1 (2020): 20651. 33244057 10.1038/s41598-020-77777-6 PMC7692499

[18] M. F. Hassan , A. N. Al‐Zurfi , M. H. Abed , and K. Ahmed , “An Effective Ensemble Learning Approach for Classification of Glioma Grades Based on Novel MRI Features,” Scientific Reports 14, no. 1 (2024): 11977. 38796531 10.1038/s41598-024-61444-1 PMC11128012

[19] M. Maskani , S. Abbasi , H. Etemad‐Rezaee , H. Abdolahi , A. Zamanpour , and A. Montazerabadi , “Grading of Gliomas by Contrast‐Enhanced CT Radiomics Features,” Journal of Biomedical Physics and Engineering 14, no. 2 (2024): 151–158. 38628893 10.31661/jbpe.v0i0.2306-1628 PMC11016825

[20] N. Haydar , K. Alyousef , U. Alanan , et al., “Role of Magnetic Resonance Imaging (MRI) in Grading Gliomas Comparable With Pathology: A Cross‐Sectional Study From Syria,” Annals of Medicine and Surgery 82 (2022): 104679. 36268388 10.1016/j.amsu.2022.104679 PMC9577635

[21] H. W. Kao , S. W. Chiang , H. W. Chung , F. Y. Tsai , and C. Y. Chen , “Advanced MR Imaging of Gliomas: An Update,” BioMed Research International 2013 (2013): 970586. 23862163 10.1155/2013/970586 PMC3686060

[22] Y. W. Park , P. Vollmuth , M. Foltyn‐Dumitru , et al., “The 2021 WHO Classification for Gliomas and Implications on Imaging Diagnosis: Part 1‐Key Points of the Fifth Edition and Summary of Imaging Findings on Adult‐Type Diffuse Gliomas,” Journal of Magnetic Resonance Imaging 58, no. 3 (2023): 677–689. 37069792 10.1002/jmri.28743

[23] L. E. Huang , “Impact of CDKN2A/B Homozygous Deletion on the Prognosis and Biology of IDH‐Mutant Glioma,” Biomedicines 10, no. 2 (2022): 246. 35203456 10.3390/biomedicines10020246 PMC8869746

[24] A. B. Khan , S. Lee , A. S. Harmanci , et al., “CXCR4 Expression Is Associated With Proneural‐to‐Mesenchymal Transition in Glioblastoma,” International Journal of Cancer 152, no. 4 (2023): 713–724. 36250346 10.1002/ijc.34329 PMC10071545

[25] J. Feng , Y. Zhang , J. Li , and X. Fan , “Roadmap Toward Subtype‐Specific Vulnerabilities in Adult Glioma,” Holistic Integrative Oncology 1, no. 1 (2022): 20.

[26] C. Pichol‐Thievend , O. Anezo , A. M. Pettiwala , et al., “VC‐Resist Glioblastoma Cell State: Vessel Co‐Option as a Key Driver of Chemoradiation Resistance,” Nature Communications 15, no. 1 (2024): 3602. 10.1038/s41467-024-47985-z PMC11058782 38684700

[27] C. Neftel , J. Laffy , M. G. Filbin , et al., “An Integrative Model of Cellular States, Plasticity, and Genetics for Glioblastoma,” Cell 178, no. 4 (2019): 835–849.e21. 31327527 10.1016/j.cell.2019.06.024 PMC6703186

[28] M. Bou Zerdan and H. I. Assi , “Oligodendroglioma: A Review of Management and Pathways,” Frontiers in Molecular Neuroscience 14 (2021): 722396. 34675774 10.3389/fnmol.2021.722396 PMC8523914

[29] P. Baumgarten , P. N. Harter , M. Tonjes , et al., “Loss of FUBP1 Expression in Gliomas Predicts FUBP1 Mutation and Is Associated With Oligodendroglial Differentiation, IDH1 Mutation and 1p/19q Loss of Heterozygosity,” Neuropathology and Applied Neurobiology 40, no. 2 (2014): 205–216. 24117486 10.1111/nan.12088

[30] A. A. Mekler , D. R. Schwartz , and O. E. Savelieva , “Genetic Discrimination of Grade 3 and Grade 4 Gliomas by Artificial Neural Network,” Cellular and Molecular Neurobiology 44, no. 1 (2023): 13. 38150033 10.1007/s10571-023-01448-z PMC11407181

[31] Y. Hoogstrate , K. Draaisma , S. A. Ghisai , et al., “Transcriptome Analysis Reveals Tumor Microenvironment Changes in Glioblastoma,” Cancer Cell 41, no. 4 (2023): 678–692.e7. 36898379 10.1016/j.ccell.2023.02.019

[32] H. Jin , Z. Yu , T. Tian , et al., “Integrative Genomic and Transcriptomic Analysis of Primary Malignant Gliomas Revealed Different Patterns Between Grades and Somatic Mutations Related to Glioblastoma Prognosis,” Frontiers in Molecular Biosciences 9 (2022): 873042. 35865002 10.3389/fmolb.2022.873042 PMC9294235

[33] T. Pienkowski , T. Kowalczyk , N. Garcia‐Romero , A. Ayuso‐Sacido , and M. Ciborowski , “Proteomics and Metabolomics Approach in Adult and Pediatric Glioma Diagnostics,” Biochimica Et Biophysica Acta. Reviews on Cancer 1877, no. 3 (2022): 188721. 35304294 10.1016/j.bbcan.2022.188721

[34] M. Guarnaccia , L. Guarnaccia , V. La Cognata , et al., “A Targeted Next‐Generation Sequencing Panel to Genotype Gliomas,” Life (Basel) 12, no. 7 (2022): 956. 35888045 10.3390/life12070956 PMC9320073

[35] L. Chen , T. Sun , J. Li , and Y. Zhao , “Identification of Hub Genes and Biological Pathways in Glioma via Integrated Bioinformatics Analysis,” Journal of International Medical Research 50, no. 6 (2022): 3000605221103976. 35676807 10.1177/03000605221103976 PMC9189557

[36] S. Lucchini , M. Constantinou , and S. Marino , “Unravelling the Mosaic: Epigenetic Diversity in Glioblastoma,” Molecular Oncology 18, no. 12 (2024): 2871–2889. 39148319 10.1002/1878-0261.13706 PMC11619803

[37] P. Rajakaruna , S. Rios , H. Elnahas , et al., “Molecular Biomarkers of Glioma,” Biomedicines 13, no. 6 (2025): 1298. 40564017 10.3390/biomedicines13061298 PMC12189432

[38] F. Li , Z. Li , H. Xu , et al., “Prediction of 1p/19q State in Glioma by Integrated Deep Learning Method Based on MRI Radiomics,” BMC Cancer 25, no. 1 (2025): 1228. 40722008 10.1186/s12885-025-14454-9 PMC12306048

[39] J. Zhang , M. Liu , Y. Fang , J. Li , Y. Chen , and S. Jiao , “TP53 R273C Mutation Is Associated With Poor Prognosis in LGG Patients,” Frontiers in Genetics 13 (2022): 720651. 35368662 10.3389/fgene.2022.720651 PMC8974296

[40] Y. Lee , C. K. Park , and S. H. Park , “Prognostic Impact of TERT Promoter Mutations in Adult‐Type Diffuse Gliomas Based on WHO2021 Criteria,” Cancers (Basel) 16, no. 11 (2024): 2032. 38893152 10.3390/cancers16112032 PMC11171308

[41] T. Hasanau , E. Pisarev , O. Kisil , N. Nonoguchi , F. Le Calvez‐Kelm , and M. Zvereva , “Detection of TERT Promoter Mutations as a Prognostic Biomarker in Gliomas: Methodology, Prospects, and Advances,” Biomedicines 10, no. 3 (2022): 728. 35327529 10.3390/biomedicines10030728 PMC8945783

[42] S. Tomoszkova , J. Skarda , and R. Lipina , “Potential Diagnostic and Clinical Significance of Selected Genetic Alterations in Glioblastoma,” International Journal of Molecular Sciences 25, no. 8 (2024): 4438. 38674026 10.3390/ijms25084438 PMC11050250

[43] H. Wang , X. Zhang , J. Liu , et al., “Clinical Roles of EGFR Amplification in Diffuse Gliomas: A Real‐World Study Using the 2021 WHO Classification of CNS Tumors,” Frontiers in Neuroscience 18 (2024): 1308627. 38595969 10.3389/fnins.2024.1308627 PMC11002900

[44] S. Chakraborty , G. Sharma , S. Karmakar , and S. Banerjee , “Multi‐OMICS Approaches in Cancer Biology: New Era in Cancer Therapy,” Biochimica et Biophysica Acta ‐ Molecular Basis of Disease 1870, no. 5 (2024): 167120. 38484941 10.1016/j.bbadis.2024.167120

[45] M. Ahmed , A. M. Semreen , W. El‐Huneidi , et al., “Preclinical and Clinical Applications of Metabolomics and Proteomics in Glioblastoma Research,” International Journal of Molecular Sciences 24, no. 1 (2022): 348. 36613792 10.3390/ijms24010348 PMC9820403

[46] H. Binder , M. Schmidt , L. Hopp , S. Davitavyan , A. Arakelyan , and H. Loeffler‐Wirth , “Integrated Multi‐Omics Maps of Lower‐Grade Gliomas,” Cancers 14, no. 11 (2022): 2797. 35681780 10.3390/cancers14112797 PMC9179546

[47] Z. Ran , J. Yang , Y. Liu , et al., “GlioMarker: An Integrated Database for Knowledge Exploration of Diagnostic Biomarkers in Gliomas,” Frontiers in Oncology 12 (2022): 792055. 36081550 10.3389/fonc.2022.792055 PMC9446481

[48] E. Chauhan , A. Sharma , M. S. Uppin , M. Kondamadugu , C. V. Jawahar , and P. K. Vinod , “IPD‐Brain: An Indian Histopathology Dataset for Glioma Subtype Classification,” Scientific Data 11, no. 1 (2024): 1403. 39702467 10.1038/s41597-024-04225-9 PMC11659595

[49] X. Deng , S. Das , H. Kaur , E. Wilson , K. Camphausen , and U. Shankavaram , “Glioma‐BioDP: Database for Visualization of Molecular Profiles to Improve Prognosis of Brain Cancer,” BMC Medical Genomics 16, no. 1 (2023): 168. 37454191 10.1186/s12920-023-01593-w PMC10350252

[50] X. Li , Y. Shao , Z. Wang , and J. Zhu , “Risk Prediction and Treatment Assessment in Glioma Patients Using SEER Database: A Prospective Observational Study,” BMJ Open 13, no. 12 (2023): e079341. 10.1136/bmjopen-2023-079341 PMC10729083 38070919

[51] W. Wu , Y. Wang , J. Xiang , et al., “A Novel Multi‐Omics Analysis Model for Diagnosis and Survival Prediction of Lower‐Grade Glioma Patients,” Frontiers in Oncology 12 (2022): 729002. 35646656 10.3389/fonc.2022.729002 PMC9133344

[52] S. Munquad and A. B. Das , “DeepAutoGlioma: A Deep Learning Autoencoder‐Based Multi‐Omics Data Integration and Classification Tools for Glioma Subtyping,” Biodata Mining 16, no. 1 (2023): 32. 37968655 10.1186/s13040-023-00349-7 PMC10652591

[53] S. Munquad , T. Si , S. Mallik , A. B. Das , and Z. Zhao , “A Deep Learning‐Based Framework for Supporting Clinical Diagnosis of Glioblastoma Subtypes,” Frontiers in Genetics 13 (2022): 855420. 35419027 10.3389/fgene.2022.855420 PMC9000988

[54] F. G. Vieira , R. Bispo , and M. B. Lopes , “Integration of Multi‐Omics Data for the Classification of Glioma Types and Identification of Novel Biomarkers,” Bioinformatics and Biology Insights 18 (2024): 11779322241249563. 38812741 10.1177/11779322241249563 PMC11135104

[55] A. Barzegar Behrooz , H. Latifi‐Navid , S. C. da Silva Rosa , et al., “Integrating Multi‐Omics Analysis for Enhanced Diagnosis and Treatment of Glioblastoma: A Comprehensive Data‐Driven Approach,” Cancers (Basel) 15, no. 12 (2023): 3158. 37370767 10.3390/cancers15123158 PMC10296097

[56] L. Li , Y. Wei , G. Shi , et al., “Multi‐Omics Data Integration for Subtype Identification of Chinese Lower‐Grade Gliomas: A Joint Similarity Network Fusion Approach,” Computational and Structural Biotechnology Journal 20 (2022): 3482–3492. 35860412 10.1016/j.csbj.2022.06.065 PMC9284445

[57] A. Kamoun , A. Idbaih , C. Dehais , et al., “Integrated Multi‐Omics Analysis of Oligodendroglial Tumours Identifies Three Subgroups of 1p/19q Co‐Deleted Gliomas,” Nature Communications 7 (2016): 11263. 10.1038/ncomms11263 PMC4838899 27090007

[58] B. Jang , D. Yoon , J. Y. Lee , et al., “Integrative Multi‐Omics Characterization Reveals Sex Differences in Glioblastoma,” Biology of Sex Differences 15, no. 1 (2024): 23. 38491408 10.1186/s13293-024-00601-7 PMC10943869

[59] M. T. Khan , B. Prajapati , S. Lakhina , et al., “Identification of Gender‐Specific Molecular Differences in Glioblastoma (GBM) and Low‐Grade Glioma (LGG) by the Analysis of Large Transcriptomic and Epigenomic Datasets,” Frontiers in Oncology 11 (2021): 699594. 34621669 10.3389/fonc.2021.699594 PMC8491982

[60] A. Braytee , S. He , S. Tang , et al., “Identification of Cancer Risk Groups Through Multi‐Omics Integration Using Autoencoder and Tensor Analysis,” Scientific Reports 14, no. 1 (2024): 11263. 38760420 10.1038/s41598-024-59670-8 PMC11101416

[61] Q. Yang , Y. Xiong , N. Jiang , F. Zeng , C. Huang , and X. Li , “Integrating Genomic Data With Transcriptomic Data for Improved Survival Prediction for Adult Diffuse Glioma,” Journal of Cancer 11, no. 13 (2020): 3794–3802. 32328184 10.7150/jca.44032 PMC7171505

[62] Q. W. Wang , Z. Zhao , Z. S. Bao , T. Jiang , and Y. J. Zhu , “Comprehensive Analysis of Multi‐Omics Data of Recurrent Gliomas Identifies a Recurrence‐Related Signature as a Novel Prognostic Marker,” American Journal of Cancer Research 11, no. 4 (2021): 1226–1246. 33948355 PMC8085869

[63] Y. Yang , F. Wang , H. Teng , et al., “Integrative Analysis of Multi‐Omics Data Reveals a Pseudouridine‐Related lncRNA Signature for Prediction of Glioma Prognosis and Chemoradiotherapy Sensitivity,” Computers in Biology and Medicine 166 (2023): 107428. 37748218 10.1016/j.compbiomed.2023.107428

[64] J. Zhang , J. Yin , L. Luo , et al., “Integrative Analysis of DNA Methylation and Transcriptome Identifies a Predictive Epigenetic Signature Associated With Immune Infiltration in Gliomas,” Frontiers in Cell and Development Biology 9 (2021): 670854. 10.3389/fcell.2021.670854 PMC8203203 34136486

[65] W. Zhang , R. Dang , H. Liu , et al., “Machine Learning‐Based Investigation of Regulated Cell Death for Predicting Prognosis and Immunotherapy Response in Glioma Patients,” Scientific Reports 14, no. 1 (2024): 4173. 38378721 10.1038/s41598-024-54643-3 PMC10879095

[66] S. R. Choi and M. Lee , “Estimating the Prognosis of Low‐Grade Glioma With Gene Attention Using Multi‐Omics and Multi‐Modal Schemes,” Biology (Basel) 11, no. 10 (2022): 1462. 36290366 10.3390/biology11101462 PMC9598836

[67] C. Toader , L. Eva , D. Costea , et al., “Low‐Grade Gliomas: Histological Subtypes, Molecular Mechanisms, and Treatment Strategies,” Brain Sciences 13, no. 12 (2023): 1700. 38137148 10.3390/brainsci13121700 PMC10741942

[68] L. Liu , S. Cui , R. Zhang , Y. Shi , and L. Luo , “MiR‐421 Inhibits the Malignant Phenotype in Glioma by Directly Targeting MEF2D,” American Journal of Cancer Research 7, no. 4 (2017): 857–868. 28469958 PMC5411793

[69] Y. Cao , H. Zhu , W. Liu , et al., “Multi‐Omics Analysis Based on Genomic Instability for Prognostic Prediction in Lower‐Grade Glioma,” Frontiers in Genetics 12 (2021): 758596. 35069679 10.3389/fgene.2021.758596 PMC8766732

[70] X. Xu , S. Zhou , Y. Tao , Z. Zhong , Y. Shao , and Y. Yi , “Development and Validation of a Two Glycolysis‐Related LncRNAs Prognostic Signature for Glioma and In Vitro Analyses,” Cell Division 18, no. 1 (2023): 10. 37355624 10.1186/s13008-023-00092-9 PMC10290322

[71] Z. Yang , X. Liu , H. Xu , et al., “Integrative Analysis of Genomic and Epigenomic Regulation Reveals miRNA Mediated Tumor Heterogeneity and Immune Evasion in Lower Grade Glioma,” Communications Biology 7, no. 1 (2024): 824. 38971948 10.1038/s42003-024-06488-9 PMC11227553

[72] R. Nassani , Y. Bokhari , and B. M. Alrfaei , “Molecular Signature to Predict Quality of Life and Survival With Glioblastoma Using Multiview Omics Model,” PLoS One 18, no. 11 (2023): e0287448. 37972206 10.1371/journal.pone.0287448 PMC10653472

[73] Y. Huang , L. Chen , Z. Zhang , et al., “Integration of Histopathological Image Features and Multi‐Dimensional Omics Data in Predicting Molecular Features and Survival in Glioblastoma,” Frontiers in Medicine 12 (2025): 1510793. 40337276 10.3389/fmed.2025.1510793 PMC12055811

[74] S. Migliozzi , Y. T. Oh , M. Hasanain , et al., “Integrative Multi‐Omics Networks Identify PKCδ and DNA‐PK as Master Kinases of Glioblastoma Subtypes and Guide Targeted Cancer Therapy,” Nature Cancer 4, no. 2 (2023): 181–202. 36732634 10.1038/s43018-022-00510-x PMC9970878

[75] X. Pan , B. Burgman , E. Wu , J. H. Huang , N. Sahni , and S. Stephen Yi , “i‐Modern: Integrated Multi‐Omics Network Model Identifies Potential Therapeutic Targets in Glioma by Deep Learning With Interpretability,” Computational and Structural Biotechnology Journal 20 (2022): 3511–3521. 35860408 10.1016/j.csbj.2022.06.058 PMC9284388

[76] P. Santamarina‐Ojeda , J. R. Tejedor , R. F. Perez , et al., “Multi‐Omic Integration of DNA Methylation and Gene Expression Data Reveals Molecular Vulnerabilities in Glioblastoma,” Molecular Oncology 17, no. 9 (2023): 1726–1743. 37357610 10.1002/1878-0261.13479 PMC10483606

[77] M. Wu , T. Wang , N. Ji , et al., “Multi‐Omics and Pharmacological Characterization of Patient‐Derived Glioma Cell Lines,” Nature Communications 15, no. 1 (2024): 6740. 10.1038/s41467-024-51214-y PMC11306361 39112531

[78] X. Ren , S. Lin , F. Guan , and H. Kang , “Glycosylation Targeting: A Paradigm Shift in Cancer Immunotherapy,” International Journal of Biological Sciences 20, no. 7 (2024): 2607–2621. 38725856 10.7150/ijbs.93806 PMC11077373

[79] Y. F. Sun , L. C. Zhang , R. Z. Niu , et al., “Predictive Potentials of Glycosylation‐Related Genes in Glioma Prognosis and Their Correlation With Immune Infiltration,” Scientific Reports 14, no. 1 (2024): 4478. 38396140 10.1038/s41598-024-51973-0 PMC10891078

[80] K. Nagai , M. Fujii , and S. Kitazume , “Protein Tyrosine Phosphatase Receptor Type Z in Central Nervous System Disease,” International Journal of Molecular Sciences 23, no. 8 (2022): 4414. 35457233 10.3390/ijms23084414 PMC9024684

[81] B. Zou , M. Li , J. Zhang , et al., “Application of a Risk Score Model Based on Glycosylation‐Related Genes in the Prognosis and Treatment of Patients With Low‐Grade Glioma,” Frontiers in Immunology 15 (2024): 1467858. 39445005 10.3389/fimmu.2024.1467858 PMC11496118

[82] M. Aldea , L. Friboulet , S. Apcher , et al., “Precision Medicine in the Era of Multi‐Omics: Can the Data Tsunami Guide Rational Treatment Decision?,” ESMO Open 8, no. 5 (2023): 101642. 37769400 10.1016/j.esmoop.2023.101642 PMC10539962

[83] Y. Ding , Z. Xu , W. Hu , P. Deng , M. Ma , and J. Wu , “Comprehensive Multi‐Omics and Machine Learning Framework for Glioma Subtyping and Precision Therapeutics,” Scientific Reports 15, no. 1 (2025): 24874. 40640380 10.1038/s41598-025-09742-0 PMC12246227

[84] Y. Yang , Z. Liu , Y. Wei , et al., “Single‐Cell Multi‐Omics Analysis Reveals Candidate Therapeutic Drugs and Key Transcription Factor Specifically for the Mesenchymal Subtype of Glioblastoma,” Cell & Bioscience 14, no. 1 (2024): 151. 39707474 10.1186/s13578-024-01332-3 PMC11662595

[85] Y. Yin , X. Fu , S. Gong , et al., “Integrative Multi‐Omics Analysis Identifies AEBP1 and EFEMP2 as Key Regulators of Immune Heterogeneity and Therapeutic Response in Glioblastoma,” Discover Oncology 16, no. 1 (2025): 1792. 41026376 10.1007/s12672-025-03500-4 PMC12484469

[86] G. Morello , V. La Cognata , M. Guarnaccia , G. Gentile , and S. Cavallaro , “Artificial Intelligence‐Driven Multi‐Omics Approaches in Glioblastoma,” International Journal of Molecular Sciences 26, no. 19 (2025): 9362. 41096631 10.3390/ijms26199362 PMC12524854

[87] M. Cieslik and A. M. Chinnaiyan , “Cancer Transcriptome Profiling at the Juncture of Clinical Translation,” Nature Reviews. Genetics 19, no. 2 (2018): 93–109. 10.1038/nrg.2017.96 29279605

[88] N. Abdelfattah , P. Kumar , C. Wang , et al., “Single‐Cell Analysis of Human Glioma and Immune Cells Identifies S100A4 as an Immunotherapy Target,” Nature Communications 13, no. 1 (2022): 767. 10.1038/s41467-022-28372-y PMC8828877 35140215

[89] I. Tirosh and M. L. Suva , “Dissecting Human Gliomas by Single‐Cell RNA Sequencing,” Neuro‐Oncology 20, no. 1 (2018): 37–43. 29016805 10.1093/neuonc/nox126 PMC5761500

[90] P. A. Cosgrove , A. H. Bild , T. H. Dellinger , B. Badie , J. Portnow , and A. Nath , “Single‐Cell Transcriptomics Sheds Light on Tumor Evolution: Perspectives From City of Hope's Clinical Trial Teams,” Journal of Clinical Medicine 13, no. 24 (2024): 7507. 39768430 10.3390/jcm13247507 PMC11677125

[91] Q. Yu , M. Jiang , and L. Wu , “Spatial Transcriptomics Technology in Cancer Research,” Frontiers in Oncology 12 (2022): 1019111. 36313703 10.3389/fonc.2022.1019111 PMC9606570

[92] K. Vandereyken , A. Sifrim , B. Thienpont , and T. Voet , “Methods and Applications for Single‐Cell and Spatial Multi‐Omics,” Nature Reviews. Genetics 24, no. 8 (2023): 494–515. 10.1038/s41576-023-00580-2 PMC9979144 36864178

[93] Q. Wang , Y. Zhi , M. Zi , et al., “Spatially Resolved Transcriptomics Technology Facilitates Cancer Research,” Advanced Science 10, no. 30 (2023): e2302558. 37632718 10.1002/advs.202302558 PMC10602551

[94] J. J. D. Moffet , O. E. Fatunla , L. Freytag , et al., “Spatial Architecture of High‐Grade Glioma Reveals Tumor Heterogeneity Within Distinct Domains,” Neuro‐Oncology Advances 5, no. 1 (2023): vdad142. 38077210 10.1093/noajnl/vdad142 PMC10699851

[95] V. M. Ravi , P. Will , J. Kueckelhaus , et al., “Spatially Resolved Multi‐Omics Deciphers Bidirectional Tumor‐Host Interdependence in Glioblastoma,” Cancer Cell 40, no. 6 (2022): 639–655.e13. 35700707 10.1016/j.ccell.2022.05.009

[96] Y. Ren , Z. Huang , L. Zhou , et al., “Spatial Transcriptomics Reveals Niche‐Specific Enrichment and Vulnerabilities of Radial Glial Stem‐Like Cells in Malignant Gliomas,” Nature Communications 14, no. 1 (2023): 1028. 10.1038/s41467-023-36707-6 PMC9950149 36823172

[97] Y. Yang , Y. Hong , K. Zhao , et al., “Spatial Transcriptomics Analysis Identifies Therapeutic Targets in Diffuse High‐Grade Gliomas,” Frontiers in Molecular Neuroscience 17 (2024): 1466302. 39530009 10.3389/fnmol.2024.1466302 PMC11552449

[98] Y. Fu , Y. Yi , Y. Shao , J. Jiang , and Q. Deng , “Single‐Cell and Spatial Transcriptomic Insights Into Glioma Cellular Heterogeneity and Metabolic Adaptations,” Frontiers in Immunology 16 (2025): 1561388. 40255400 10.3389/fimmu.2025.1561388 PMC12006195

[99] J. Zhao , “Network Neurosurgery,” Chinese Neurosurgical Journal 9, no. 1 (2023): 4. 36732790 10.1186/s41016-023-00317-4 PMC9893634

[100] M. Serban , C. Toader , and R. A. Covache‐Busuioc , “Precision Neuro‐Oncology in Glioblastoma: AI‐Guided CRISPR Editing and Real‐Time Multi‐Omics for Genomic Brain Surgery,” International Journal of Molecular Sciences 26, no. 15 (2025): 7364. 40806492 10.3390/ijms26157364 PMC12346968

[101] A. Omuro and L. M. DeAngelis , “Glioblastoma and Other Malignant Gliomas: A Clinical Review,” Journal of the American Medical Association 310, no. 17 (2013): 1842–1850. 24193082 10.1001/jama.2013.280319

[102] W. Xing and C. Zeng , “An Integrated Transcriptomic and Computational Analysis for Biomarker Identification in Human Glioma,” Tumour Biology 37, no. 6 (2016): 7185–7192. 26663173 10.1007/s13277-015-4585-6

[103] J. S. Wekesa and M. Kimwele , “A Review of Multi‐Omics Data Integration Through Deep Learning Approaches for Disease Diagnosis, Prognosis, and Treatment,” Frontiers in Genetics 14 (2023): 1199087. 37547471 10.3389/fgene.2023.1199087 PMC10398577

[104] R. Deleanu , L. C. Ceafalan , and A. Dricu , “Transcriptomic Crosstalk Between Gliomas and Telencephalic Neural Stem and Progenitor Cells for Defining Heterogeneity and Targeted Signaling Pathways,” International Journal of Molecular Sciences 22, no. 24 (2021): 13211. 34948008 10.3390/ijms222413211 PMC8703403

[105] P. Sledzinska , M. G. Bebyn , J. Furtak , J. Kowalewski , and M. A. Lewandowska , “Prognostic and Predictive Biomarkers in Gliomas,” International Journal of Molecular Sciences 22, no. 19 (2021): 10373. 34638714 10.3390/ijms221910373 PMC8508830

[106] Y. Ozaki , P. Broughton , H. Abdollahi , H. Valafar , and A. V. Blenda , “Integrating Omics Data and AI for Cancer Diagnosis and Prognosis,” Cancers (Basel) 16, no. 13 (2024): 2448. 39001510 10.3390/cancers16132448 PMC11240413

[107] Y. J. Heo , C. Hwa , G. H. Lee , J. M. Park , and J. Y. An , “Integrative Multi‐Omics Approaches in Cancer Research: From Biological Networks to Clinical Subtypes,” Molecules and Cells 44, no. 7 (2021): 433–443. 34238766 10.14348/molcells.2021.0042 PMC8334347

